# Production of Salvianic Acid A from l-DOPA via Biocatalytic Cascade Reactions

**DOI:** 10.3390/molecules27186088

**Published:** 2022-09-18

**Authors:** Ke Shun Hu, Chong Le Chen, Huan Ru Ding, Tian Yu Wang, Qin Zhu, Yi Chen Zhou, Jia Min Chen, Jia Qi Mei, Sheng Hu, Jun Huang, Wei Rui Zhao, Le He Mei

**Affiliations:** 1 School of Biotechnology and Chemical Engineering, NingboTech University, Ningbo 315100, China; 2 Department of Chemical and Biological Engineering, Zhejiang University of Science and Technology, Hangzhou 310023, China; 3 Hangzhou Huadong Medicine Group Co. Ltd., Hangzhou 310011, China; 4 Department of Chemical and Biological Engineering, Zhejiang University, Hangzhou 310027, China; 5 Jinhua Advanced Research Institute, Jinhua 321019, China

**Keywords:** salvianic acid A, l-DOPA, membrane-bound l-amino acid deaminases, biocatalysis, molecular biology, biological engineering

## Abstract

Salvianic acid A (SAA), as the main bioactive component of the traditional Chinese herb *Salvia miltiorrhiza*, has important application value in the treatment of cardiovascular diseases. In this study, a two-step bioprocess for the preparation of SAA from l-DOPA was developed. In the first step, l-DOPA was transformed to 3,4-dihydroxyphenylalanine (DHPPA) using engineered *Escherichia coli* cells expressing membrane-bound L-amino acid deaminase from *Proteus vulgaris.* After that, the unpurified DHPPA was directly converted into SAA by permeabilized recombinant *E. coli* cells co-expressing d-lactate dehydrogenase from *Pediococcus acidilactici* and formate dehydrogenase from *Mycobacterium vaccae* N10. Under optimized conditions, 48.3 mM of SAA could be prepared from 50 mM of l-DOPA, with a yield of 96.6%. Therefore, the bioprocess developed here was not only environmentally friendly, but also exhibited excellent production efficiency and, thus, is promising for industrial SAA production.

## 1. Introduction

Salvianic acid A [SAA, (*R*)-(+)-3,4-dihydroxyphenyllactic acid], also known as danshensu, is the main bioactive ingredient of the traditional Chinese herb *Salvia miltiorrhiza* (danshen) [1]. SAA is well-known for its distinguished antioxidant activity [1,2] and pharmacological activities, such as improving cerebral blood flow [3], the inhibition of platelet activation and arterial thrombosis [4], and its anti-atherosclerosis [5], anticancer [6], and anti-inflammatory effects [7]. Recently, SAA showed promise in applications for alleviating alcohol-induced acute liver damage [8] and myocardial ischemia injury [9], and it was also reported to confer substantial neuroprotection against Tat-induced neurotoxicity [10]. SAA derivatives, such as salvianolic acids B and A, and conjugates of SAA with cysteine also exhibited excellent cardiovascular-protective effects with promising application in the pharmaceutical and food industries [11,12,13,14].

Traditionally, SAA has been isolated from the dried root of *S. miltiorrhiza* by a water-extraction process [15,16]. However, the amount of SAA in the crude root of *S. miltiorrhiza* is quite low (0.045%) [17]. In addition, the growth of *S. miltiorrhiza* is affected by region and climate, making production unpredictable and insufficient to meet the growing market demand, thereby restricting the large-scale application of SAA from *S. miltiorrhiza*. Although several chemical methods for SAA synthesis have been developed, these methods also suffer from intractable enantioselectivities, complicated procedures, and environmental pollution [18,19,20]. Thus, an enzymatic method for SAA production was developed as a “green” alternative by converting 3,4-dihydroxyphenylalanine (DHPPA), an α-keto acid and the direct precursor of SAA, via d-type NADH-dependent dehydrogenase (especially d-lactate dehydrogenase (d-LDH)) [21,22]. However, the existing methods for DHPPA biosynthesis either involve a multi-step process that requires harsh chemicals [21] or require expensive catalysts [22]. In addition, high-priced NADH is required as a co-enzyme for NADH-dependent dehydrogenases to convert DHPPA to SAA. Therefore, there is an urgent need to develop a biotransformation system that can circumvent these disadvantages for SAA biosynthesis.

The amino acid l-DOPA structurally resembles DHPPA and can also be converted to DHPPA through enzymatic deamination. In addition, l-DOPA can be produced through a relatively easily and cheap process [23,24]; thus, it is a promising starting material for the biological preparation of SAA. Therefore, we developed a biotransformation system to prepare SAA using l-DOPA as the starting material and the following synthesis route: an initial conversion of l-DOPA to DHPPA and a subsequent reduction of DHPPA to SAA.

To convert l-DOPA (amino acids) to DHPPA (α-keto acids), four kinds of enzymes, including amino acid dehydrogenase (ADH), amino acid transferase (AT), l-amino acid oxidase (l-AAO), and membrane-bound l-amino acid deaminase (ml-AAD), can be used to conduct this reaction. Among them, the AT-mediated reaction is reversible and requires another α-keto acid as an amino group acceptor [25]; ADH also catalyzes a reversible reaction [26]. Although the l-AAO-mediated deamination reaction is irreversible, hydrogen peroxide (H_2_O_2_) is produced during the reaction, resulting in the destruction of nascent α-keto acids and the denaturation of the enzymes [27,28]. In addition, the generation of H_2_O_2_ poses a challenge to l-AAO overexpression, which usually leads to insoluble protein formation or low expression levels [28]. Therefore, none of these three enzymes is suitable for the mass production of DHPPA from an economical point of view. Alternatively, ml-AAD, which is anchored on the outer side of the cytomembrane, catalyzes the irreversible deamination of natural l-amino acids to generate the respective α-keto acids, ammonia, and H_2_O [28,29]. Compared with the above three enzymes, ml-AAD neither requires co-enzymes or amino receptors nor produces H_2_O_2_ as a byproduct of the reaction. Moreover, ml-AAD can be easily overexpressed in expression hosts compare with l-AAO [28,30], making it the best choice for the preparation of DHPPA from l-DOPA. In addition, there is no permeability barrier of the cell membrane against the substrates and products of the ml-AAD catalytic system because the enzyme is anchored on the outer side of the cytomembrane [28,29].

The BRENDA enzyme database reports that NADH-dependent d-LDH from lactic acid bacteria strains and hydroxyphenylpyruvate reductase (HPPR) from *Coleus blumei* can convert DHPPA to SAA. However, to use NADH-dependent enzymes, expensive NADH needs to be supplied as a co-enzyme, which is oxidized into NAD^+^. To solve the problem of NADH supply, an NADH regeneration system can be introduced. Currently, glucose dehydrogenase (GDH) and formate dehydrogenase (FDH) are the two most widely used NADH regeneration systems [31,32]. Compared with GDH, FDH is preferable for whole-cell transformation because it uses inexpensive formate as a cosubstrate and oxidizes formate to carbon dioxide and H_2_O without any other environmentally unfriendly coproducts [32].

**Figure 1:**
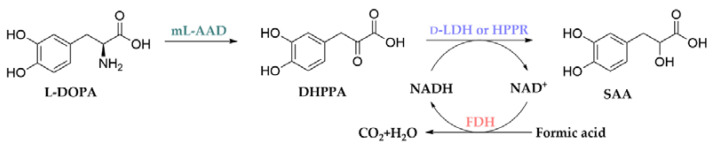
Schematic of the synthesis of salvianic acid A (SAA) from l-DOPA. DHPPA: 3,4-dihydroxyphenylalanine; ml-AAD: membrane-bound l-amino acid deaminases; d-LDH: d-lactate dehydrogenase; HPPR: hydroxyphenylpyruvate reductase; FDH: formate dehydrogenase.

Therefore, we select ml-AAD, d-LDH or HPPR, and FDH to conduct a two-step biocatalytic system to produce SAA from l-DOPA. In the first step, recombinant *Escherichia coli* cells expressing ml-AAD are employed to deaminize l-DOPA to form DHPPA. In the second step, recombinant *E. coli* cells co-expressing d-LDH and FDH or co-expressing HPPR and FDH are used to convert DHPPA in the raw reaction solution to SAA. (Figure 1).

## 2. Results and Discussion

### 2.1. Biotransformation of l-DOPA to DHPPA by BL21(DE3)-pET-28a-mlaad

**Figure 2:**
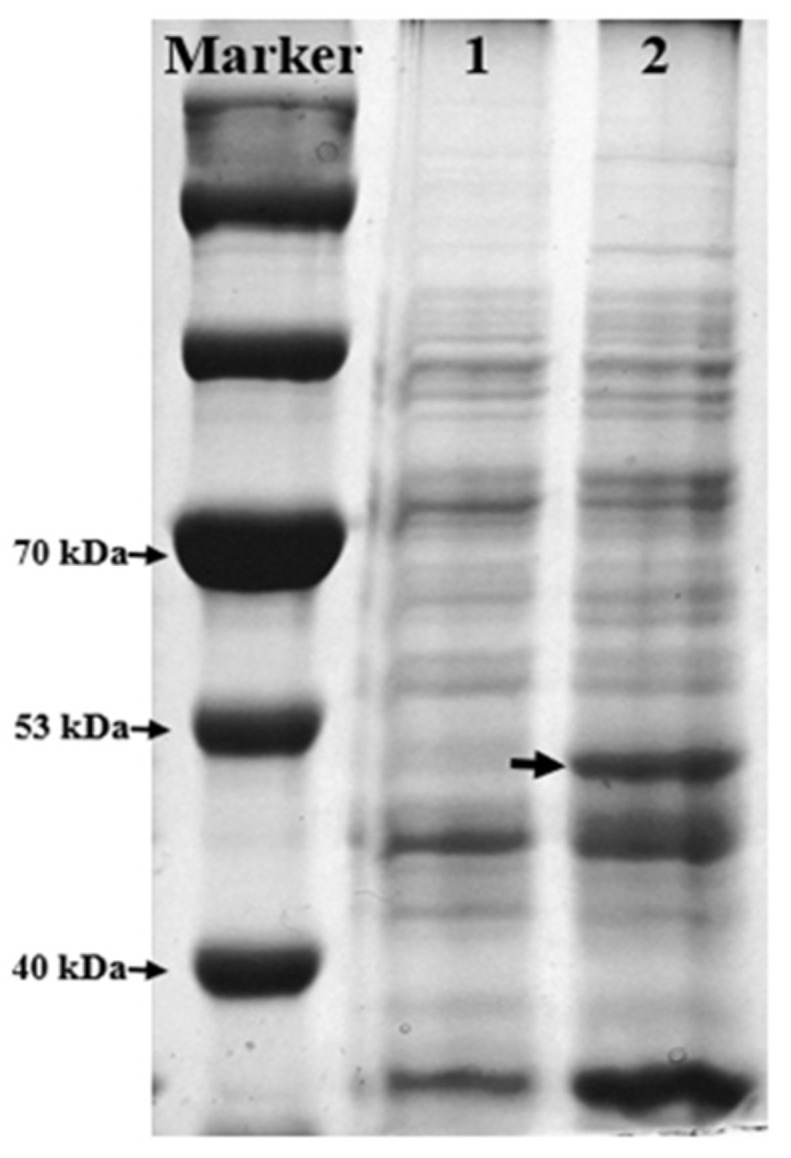
SDS-PAGE analysis of ml-AAD expression in BL21(DE3)-pET-28a-*mlaad*. *Lane 1*, membrane fractions of BL21(DE3)-pET28a (control); *lane 2*, membrane fractions of BL21(DE3)-pET-28a-*mlaad*; The same amounts of cells were loaded in lane 1 and lane 2. Bands indicated by arrow, recombinant ml-AAD; the theoretical protein size was 51.5 kDa. Both BL21(DE3)-pET-28a-*mlaad* and the control were induced using 0.5 mM IPTG at 28 °C and 150 rpm for 6 h.

Given that each ml-AAD has its own substrate spectrum [33], selecting the most suitable ml-AAD for converting l-DOPA to DHPPA is an important first step in the development of a new enzymatic synthesis method. Among the reported ml-AADs, ml-AAD from *P. vulgaris* shows higher activity toward hydrophobic amino acids, such as l-tyrosine and l-histidine [33,34]. Considering the structural similarity between l-DOPA and tyrosine, which both contain a phenol structure, we speculated that the ml-AAD from *P. vulgaris* might also have good activity toward l-DOPA. We detected the expression level of ml-AAD in *E. coli* (because ml-AAD is a membrane-bound protein, the membrane fraction of recombinant *E. coli* cells was detected) and the ml-AAD catalytic activity toward l-DOPA. The results showed that recombinant ml-AAD was overexpressed in *E. coli* (Figure 2), and its activity toward l-DOPA (50.7 μmol·min^−1^·g^−^^1^, determination condition: cell biomass: 0.42 g·L^−1^, 10 mM l-DOPA and pH 7.5) was 44% lower than its activity toward tyrosine; however, its catalytic activity toward l-DOPA was much higher than its activities toward most non-aromatic amino acids, as reported by Hossain (2014) [28] and Baek (2011) [33], which was consistent with our prediction. Therefore, we prepared a recombinant *E. coli* whole-cell biocatalyst expressing ml-AAD from *P. vulgaris* (BL21(DE3)-pET-28a-*mlaad*) to convert l-DOPA.

**Figure 3:**
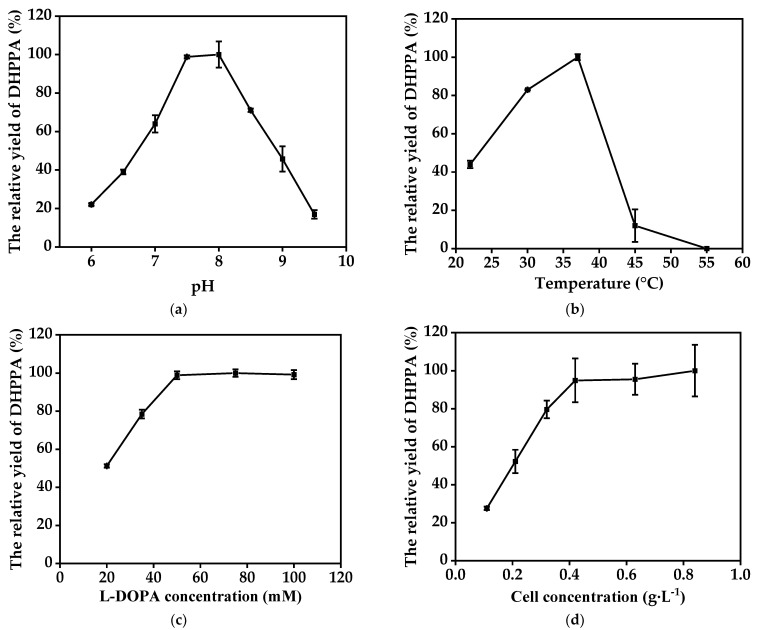
(**a**) Effects of pH on the relative yield of DHPPA (reactions were performed at 37 °C in reaction mixture comprised of 0.42 g·L^−1^ recombinant cells and 20 mM l-DOPA (pH 5–10); the DHPPA yield at pH 7.5 was set as 100%); (**b**) effects of temperature on the relative yield of DHPPA (reactions were performed at 22–55 °C in reaction mixture comprised of 0.42 g·L^−1^ recombinant cells and 20 mM l-DOPA (pH 7.5); the DHPPA yield at 37 °C was set as 100%); (**c**) effects of substrate concentration on the relative yield of DHPPA (reactions were performed at 37 °C in reaction mixture comprised of 0.42 g·L^−1^ recombinant cells and 20-100 mM l-DOPA (pH 7.5); the DHPPA yield at 50 mM l-DOPA was set as 100%); (**d**) effects of cell concentration on the relative yield of DHPPA (reactions were performed at 37 °C in reaction mixture comprised of 0.11–0.84 g·L^−1^ recombinant cells and 50 mM l-DOPA (pH 7.5); the DHPPA yield at 0.84 g·L^−1^ cell concentration was set as 100%). Data represent the means ± SD from three independent determinations.

We next examined the effect of the bioconversion conditions on DHPPA production. Because l-DOPA is readily oxidized to melanin, especially in an alkaline environment, 17 mM ascorbic acid was added to the reaction system to inhibit this side reaction. As shown in Figure 3a, the highest productivity of DHPPA was obtained in the ml-AAD catalytic reaction between pH 7.5 and 8.0; when the pH was below 7.5, the DHPPA productivity sharply decreased. Figure 3b shows the effect of reaction temperatures ranging from 20 °C to 55 °C on the productivity. The highest productivity of DHPPA was observed at 37 °C. Moreover, the DHPPA productivity increased with increasing concentration of l-DOPA in the range of 20–50 mM, and it could not be enhanced at the higher l-DOPA concentrations tested (Figure 3c). Considering that l-DOPA is easily oxidized, it was not suitable to add too much l-DOPA in the initial reaction solution; therefore, we used 50 mM substrates for further evaluations (if a higher l-DOPA concentration is needed, a feeding strategy can be adopted). The DHPPA yield initially increased with increasing cell concentration and then reached a plateau (Figure 3d). The production of DHPPA could not be effectively enhanced at biocatalyst concentrations above 0.42 g·L^−1^, which was mainly due to substrate and catalyst saturation. Thus, the optimal conditions were determined as follows: 0.42 g·L^−1^ cell biomass, 50 mM l-DOPA concentration, 37 °C, and pH 7.5.

**Figure 4:**
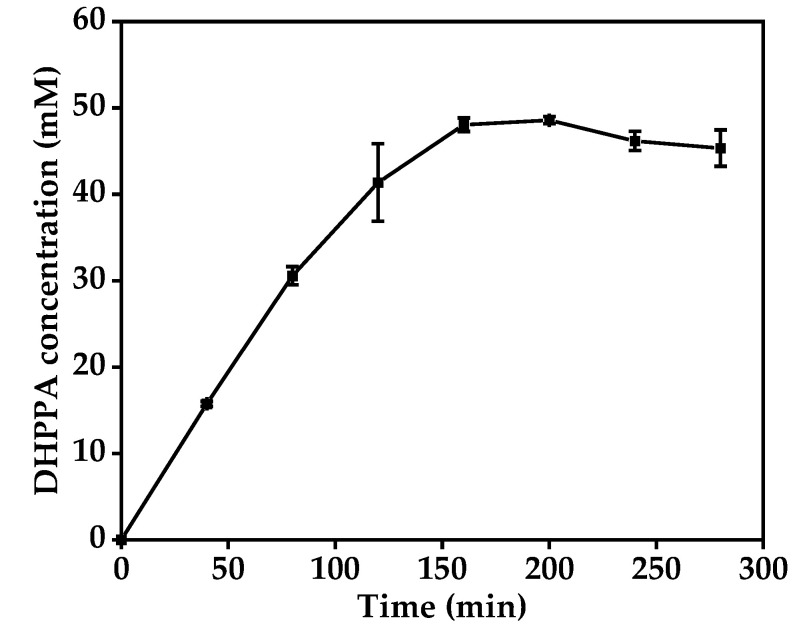
Time profile for the production of DHPPA from l-DOPA using BL21(DE3)-pET-28a-*mlaad* whole-cell catalysts under optimal conditions. Data represent the means ± SD from three independent determinations.

Next, we performed the biotransformation of l-DOPA to DHPPA under the optimized conditions. As shown in Figure 4, the highest yield of DHPPA (48.59 mM) was obtained after 160 min, with a conversion rate of 97.18%. With further increase in the reaction time, the reaction mixture gradually became brown, which was mainly due to the exhaustion of the 17 mM ascorbic acid added in the initial reaction system. Without ascorbic acid protection, product degradation was observed after 200 min. This indicated that more ascorbic acid should be added when using greater concentrations of l-DOPA (>50 mM). Thus, 160 min was determined to be an ideal operation time for DHPPA production with 50 mM l-DOPA in our reaction system. In addition, we determined the reusability of BL21(DE3)-pET-28a-*mlaad* and found that the DHPPA yield with BL21(DE3)-pET-28a-*mlaad* was only about 36% of the original yield in second cycle, indicating that the BL21(DE3)-pET-28a-*mlaad* cells could not be reused in our reaction system.

### 2.2. Biotransformation of DHPPA to SAA

**Figure 5:**
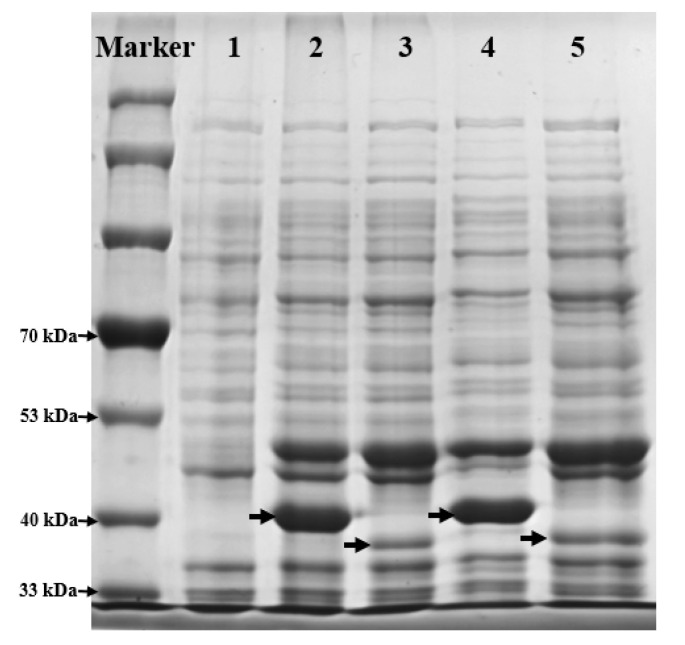
SDS-PAGE analysis of total cell lysates and the soluble constituents of BL21(DE3)-pETDuet-*dldh*-*fdh* and BL21(DE3)-pETDuet-*hppr*-*fdh*. Total cell lysates of BL21(DE3) were used as control (*lane 1*); total cell lysates and soluble constituents of BL21(DE3)-pETDuet-*dldh*-*fdh* were loaded in *lanes 2* and *4*, respectively; total cell lysates and soluble constituents of BL21(DE3)-pETDuet-*hppr*-*fdh* were loaded in *lanes 3* and *5*, respectively; bands indicated by arrows in *lanes 2* and *4* were recombinant D-LDH; the theoretical protein size was 37.2 kDa; bands indicated by arrows in *lanes 3* and *5* were recombinant HPPR; the theoretical protein size was 35.4 kDa. The same amounts of cells were loaded in all lanes. All cells were induced using 0.5 mM IPTG at 28 °C and 150 rpm for 6 h.

d-LDH from some lactic acid bacteria strains and HPPR from *C. blumei* have been reported to reduce the α-keto group of DHPPA to a hydroxy group, thereby achieving the conversion of DHPPA to SAA [21,35]. To select a more efficient biotransformation system, we compared the bioconversion ability of SAA from DHPPA using HPPR from *C. blumei* and d-LDH from *P. acidilactici* DSM 20284. Since both HPPR and d-LDH are NADH-dependent enzymes, FDH was introduced into the two enzymatic systems as a cofactor for NADH regeneration. Therefore, we co-expressed FDH from *M. vaccae* N10 with HPPR (BL21(DE3)-pETDuet-*hppr*-*fdh*) and d-LDH (BL21(DE3)-pETDuet-*dldh*-*fdh*), respectively. By incubating the two recombinant *E. coli* cells (2.48 g·L^−1^) in the reaction mixture (20 mM DHPPA, 40 mM sodium formate, pH 6) for 1 h, 2.4 mM SAA was produced by BL21(DE3)-pETDuet-*dldh*-*fdh,* while BL21(DE3)-pETDuet-*hppr*-*fdh* did not exhibit obvious activity. We used SDS-PAGE to detect the expression level of HPPR in BL21(DE3)-pETDuet-*hppr*-*fdh* and found that, although its expression level was much lower than that of d-LDH in BL21(DE3)-pETDuet-*dldh*-*fdh*, the HPPR was mostly expressed in its soluble form (Figure 5). In addition, no obvious HPPR activity could be detected, even in the soluble constituents of BL21(DE3)-pETDuet-*hppr*-*fdh* cell lysates. Therefore, we inferred that the low SAA production with BL21(DE3)-pETDuet-*hppr*-*fdh* was mainly caused by the low activity of HPPR in *E. coli*. Therefore, the recombinant strain BL21(DE3)-pETDuet-*dldh*-*fdh* was selected for further study. In addition, without the addition of sodium formate (i.e., without NADH regeneration), the production of SAA from DHPPA catalyzed with BL21(DE3)-pETDuet-*dldh*-*fdh* was much lower than that observed in the presence of sodium formate, which indicated that the regeneration system was essential for d-LDH activity.

**Figure 6:**
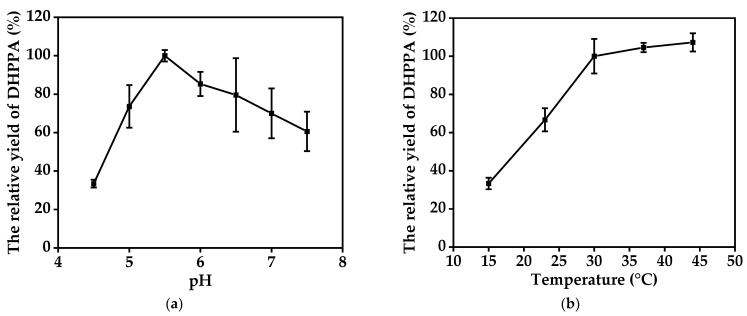
(**a**) Effects of pH on SAA production yield (the SAA yield at pH 5.5 was set as 100%). (**b**) Effects of temperature on SAA production yield (the SAA yield at 44 °C was set as 100%). Data represent the means ± SD from three independent determinations.

During our preliminary experiments, we found that the cell-bound activity of BL21(DE3)-pETDuet-*dldh*-*fdh* was much lower than the activity of the BL21(DE3)-pETDuet-*dldh*-*fdh* cell lysates. Thus, we permeabilized BL21(DE3)-pETDuet-*dldh*-*fdh* with hexane prior to the reaction. After permeabilization, the SAA yield in 1 h with the treated BL21(DE3)-pETDuet-*dldh*-*fdh* was enhanced 8.7-fold more than that of the untreated cells (the SAA yield in 1 h with 2.48 g·L^−1^ permeabilized BL21(DE3)-pETDuet-*dldh*-*fdh* was 14 mM). To increase the efficiency of SAA production, the biocatalytic conditions of the coupling system were further optimized. The SAA production rate increased with increasing pH from 4.5 to 5.5 and then decreased at higher pH values; thus, the maximal SAA conversion rate from DHPPA was obtained at pH 5.5 (Figure 6a). The SAA yield initially increased with increasing temperature and then reached a plateau in the tested range (Figure 6b). The production of SAA did not effectively change when the temperature was higher than 30 °C, which was likely due to mutual effects between d-LDH and FDH. For the sake of minimizing energy consumption, 30 °C and pH 5.5 were adopted as the optimal conditions to prepare SAA from the conversion of DHPPA.

### 2.3. Two-Step Catalytic Synthesis of SAA from l-DOPA

**Figure 7:**
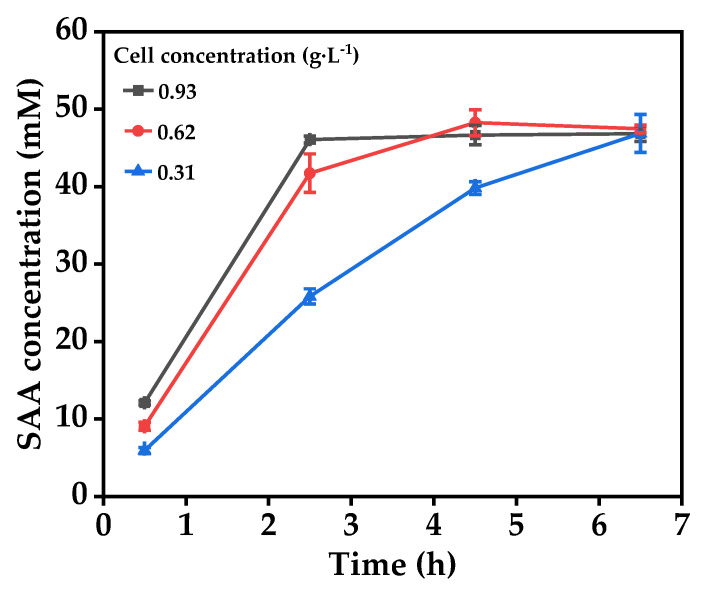
Effect of permeabilized BL21(DE3)-pETDuet-*dldh*-*fdh* cell concentration on SAA.

DHPPA was prepared with BL21(DE3)-pET-28a-*mlaad* under the optimized deamination conditions described above, resulting in 48.6 mM DHPPA from 50 mM l-DOPA (Figure 4). Next, the recombinant cells were removed from the reaction solution by centrifugation, 100 mM sodium formate and 10 mM NAD^+^ were added, and the reaction pH was adjusted to 5.5. Subsequently, 0.31–0.93 g·L^−1^ permeabilized BL21(DE3)-pETDuet-*dldh*-*fdh* was added to the first step’s reaction solution to start the conversion of DHPPA to SAA. As shown in Figure 7, the yields of SAA from DHPPA in our experimental ranges all reached more than 97.7%, and the SAA production rate was accelerated with increasing cell concentration. When the cell concentration was above 0.62 g·L^−1^, DHPPA could almost be completely converted to SAA within 4.5 h. With a cell concentration of 0.31 g·L^−1^, the SAA conversion rate from DHPPA reached over 97.7% after 6.5 h.

Overall, in our developed two-step biotransformation process, l-DOPA was efficiently deaminized to DHPPA with a high yield of 97.7% in ml-AAD bioconversion, and then the DHPPA was effectively converted to SAA with permeabilized recombinant *E. coli* cells co-expressing d-LDH and FDH. The total yield of SAA from l-DOPA could reach approximately 96.5% using the two-step biocatalytic reaction under the optimum reaction conditions. In addition, we determined the reusability of BL21(DE3)-pETDuet-*dldh*-*fdh* using 0.31 g·L^−1^ cells and found that the SAA yield with the biocatalyst was above 85% of the original yield, even in fifth cycle, indicating that the permeabilized BL21(DE3)-pETDuet-*dldh*-*fdh* cells had excellent reusability.

Several synthetic methods involving the biological steps for SAA production have been reported [21,22]. Yang et al. [23] developed a chemoenzymatic process to synthesize SAA. In the method, the intermediate DHPPA was prepared from 3,4-dihydroxybenzaldehyde and acetyl glycine through an initial Erlenmeyer condensation ring-opening reaction; subsequently, 4.7 mM SAA was obtained after a 24 h enzymatic reduction of 5.1 mM chemical-prepared DHPPA with 20 g/L resting cells of *Pediococcus acidilactici*, with an overall yield of 69. 4% [23]. However, the production process of DHPPA involved harsh chemicals and multistep reactions, and the cell preparations in that chemoenzymatic method for DHPPA conversion were not efficient. Additionally, a completely enzymatic method for SAA synthesis was developed by Findrik et al. [21]. In the process, DHPPA was prepared through the oxidative deamination of l-DOPA using l-amino acid oxidase (l-AAO) from the snake venom *Crotalus adamanteus* (catalase from beef liver was also added to the reaction system to prevent the oxidative decarboxylation of DHPPA mediated by the generated H_2_O_2_ in the deamination reaction); subsequently, the intermediate DHPPA was converted to SAA using purified d-LDH from *Lactobacillus leishmannii*, with a volumetric productivity of 93.06 mg·L^−1^·d^−1^ [24]. Although the method was environmentally friendly, it had some disadvantages. Specifically, the production efficiency of the method was not satisfactory. In addition, the H_2_O_2_ generated in the l-AAO-catalyzed reaction could denature the enzyme and newly produced DHPPA; thus, extra catalase needed to be added to eliminate this negative effect, which sharply increased the production costs [24]. Moreover, l-AAOs were difficult to be produced through overexpression due to the generated H_2_O_2_, which made large-scale production more difficult. In our study, we used ml-AAD from *P. vulgaris* to convert l-DOPA to DHPPA rather than l-AAO. Although both l-AAOs and ml-AADs belong to the category EC1.4.3.2, they exhibit different modes of actions. l-AAOs rely on a typical oxidative deamination mechanism to produce α-keto acids, along with equimolar amounts of ammonia and H_2_O_2_. In contrast, ml-AADs are associated with the electron transport chain on the bacterial cell membrane and adopt a noncanonical catalytic mechanism in which the electrons produced in deamination reactions are eventually transferred to cytochrome oxidases to reduce O_2_ to H_2_O [36]; thus, H_2_O_2_ is not produced in ml-AAD-driven reactions. Without H_2_O_2_ production, the extra catalase was not needed in our developed systems, unlike in the method reported by Findrik et al. [21]. In addition, without H_2_O_2_ generation in ml-AAD reactions, ml-AADs can be more easily overexpressed in hosts than l-AAOs [28,29], which sharply reduces the costs of preparing biocatalysts compared with Findrik’s method [21]. Therefore, ml-AADs present a deamination process that is more practical, economical, and suitable for industrial use. Moreover, we used a whole-cell catalyst rather than purified enzymes in the reactions, which could be easily prepared and separated from the reaction system, and the volumetric productivity for our process was 24.62 g·L^−1^·d^−1^ when 0.31 g·L^−1^ permeabilized BL21(DE3)-pETDuet-*dldh*-*fdh* cells if only the reaction time in the two steps was considered. More importantly, the method developed herein exhibited an excellent production efficiency and demonstrated good industrial application prospects.

## 3. Materials and Methods

### 3.1. Chemicals

The strains *E. coli* BL21(DE3) and *E. coli* DH5α were purchased from TransBionovo Co., Ltd. (Beijing, China). SAA standard was obtained from Shanghai Yuanye Biotech Co., Ltd. (Shanghai, China). l-DOPA was purchased from Aladdin Industrial Corporation Technology (Shanghai, China). DHPPA was purchased from Yantai Kaibo Pharmaceutical Co., Ltd. (Yantai, China). Sodium formate and vitamin C were provided by Shanghai Sangon Biotech Co., Ltd. (Shanghai, China). All other chemicals were of analytical grade or higher.

### 3.2. Microorganisms and Shake-Flask Fermentation

For the expression of membrane-bound ml-AAD, the *P. vulgaris* ml-AAD gene (GenBank accession no. AB030003.1) was amplified using polymerase chain reaction (PCR) and inserted into the pET-28a plasmid between the *Nco* I and *Xho* I restriction sites, yielding plasmid pET-28a-*mlaad* [34]. For the co-expression of FDH from *Mycobacterium vaccae* N10 and d-LDH from *Pediococcus acidilactici* DSM 20284, the FDH gene (GenBank accession no. AB072394.1) was codon-optimized, synthesized, and inserted into multiple cloning sites-2 of the pETDuet-1 vector between the *Nde* I and *Xho* I sites, generating plasmid pETDuet-*fdh*. The d-LDH gene (GenBank accession no. AEEG01000002) was PCR-amplified with the forward primer LDHF (5′-TACCCCATGGCCATGAAGATTATTGCTTATG-3′) and reverse primer LDHR (5′-TCGAGCGGCCGCTTAGTCAAACTTAACTTCATT-3′) and inserted into the *Nco* I and *Not* I sites of the expression vector pETDuet-*fdh* to obtain the co-expression plasmid pETDuet*-dldh-fdh.* For the co-expression of FDH from *M. vaccae* N10 and the hydroxyphenylpyruvate reductase (HPPR) gene from *Coleus blumei*, the HPPR gene (GenBank accession no. AJ507733.2) was codon-optimized (Supplementary Materials), synthesized, and inserted into multiple cloning sites-1 of the pETDuet-*fdh* vector between the *Bam*H I and *Hin*d III sites, generating the co-expression plasmid pETDuet*-sddh- fdh*. All recombinant cells were constructed by transforming the corresponding plasmids into *E. coli* BL21(DE3).

The recombinant strain was inoculated in 5 mL lysogeny broth (LB) medium and cultivated on a rotary shaker at 37 °C and 200 rpm overnight. Subsequently, a 2% seed culture was inoculated into 50 mL LB medium in a 250 mL flask and cultured on a rotary shaker (37 °C, 200 rpm) until the optical density at 600 nm (OD_600_) reached 0.6–0.8. Isopropyl-β-d-1-thiogalactopyranoside (IPTG) was then added to the culture at a final concentration of 0.5 μM to induce recombinant protein expression under incubation at 28 °C at 150 rpm for 6 h. The recombinant *E. coli* cells were harvested from the culture medium by centrifugation at 10,000× *g* for 1 min at 4 °C, and the cell pellets were washed with sodium phosphate buffer (0.2 M, pH 7.5). Induced cells were disrupted by sonication. Cell lysates were separated into supernatant and precipitated fractions by centrifugation at 10,000× *g* for 10 min at 4 °C. The membrane fractions of induced cells were prepared by using a bacterial membrane protein extraction kit (BestBio Co., Shanghai, China). Protein samples from each fraction were analyzed using SDS-PAGE.

### 3.3. Optimization of the ml-AAD-Catalyzed Reaction

For optimization of all the variables in the reaction system, an ml-AAD-mediated reaction was performed with a reaction mixture of 1 mL comprising 0.2 mM sodium phosphate buffer, BL21(DE3)-pET-28a-*mlaad* cells, l-DOPA, and 17 mM vitamin C on a thermoshaker incubator at a rotation speed of 800 rpm for 1 h. Reactions were then performed under a range of pH values (6.0–9.5), temperatures (20–55 °C), cell concentrations (0.1–0.85 g·L^−^^1^ dry cell weight (DCW)), and l-DOPA concentrations (20–100 mM). The reaction was stopped by adding an equal volume of 1 M HCl, and the DHPPA concentration in the reaction solution was determined using high-performance liquid chromatography (HPLC), as described below. 

For DHPPA production, the biotransformation was performed with 0.42 g·L^−1^ BL21(DE3)-pET-28a*-mlaad* cells in 10 mL reaction solution with 50 mM l-DOPA under the optimized conditions at an agitation speed of 800 rpm.

### 3.4. Biotransformation of DHPPA to SAA

To break through the cell envelope barrier against the diffusion of substrates and products, the BL21(DE3)-pETDuet*-dldh-fdh* cells were permeabilized with 1% hexane (*v*/*v*) for 10 min before initiating the reactions. The effects of pH and temperature on the SAA conversion rates were investigated by incubating 0.62 g·L^−1^ (DCW) permeabilized BL21(DE3)-pETDue*t-dldh-fdh* cells in a reaction mixture containing 0.2 M sodium phosphate buffer, 20 mM DHPPA, 40 mM sodium formate (the molar ratio of DHPPA to sodium formate was maintained at 1:2), and 10 mM NAD^+^ for 1 h. For temperature optimization, the reactions were conducted at pH 6.0 with a range of temperatures from 15 °C to 44 °C. For pH optimization, the reactions were performed at 30 °C in the pH range from 4.5 to 7.5. The reaction mixture was heated to 100 °C for 5 min to terminate the reactions, and the SAA concentration in the produced supernatants was quantitatively determined using HPLC, as described below.

### 3.5. Production of SAA from l-DOPA Using a Two-Step Cascade Reaction

The first step in the reaction for preparing DHPPA from l-DOPA was conducted using 0.42 g·L^−1^ BL21(DE3)-pET-28a-*mlaad* cells, 50 mM l-DOPA, and 17 mM vitamin C at pH 7.5 and 37 °C for 160 min. Subsequently, BL21(DE3)-pET-28a-*mlaad* cells were removed from the reaction solution by centrifugation, 100 mM sodium formate and 10 mM NAD^+^ were added to the reaction solution, and the pH was adjusted to 5.5. The reaction solutions were mixed with different concentrations of permeabilized BL21(DE3)-pETDuet-*dhd*-*fdh* cells and incubated on a thermoshaker at 800 rpm and 30 °C for the conversion of DHPPA to SAA.

### 3.6. Analysis Test Method

DHPPA and SSA present in the reaction mixture were quantified using HPLC. When preparing DHPPA, the concentration of DHPPA was analyzed using a Shimadzu 2030 HPLC system equipped with a Hypersil ODS2 C18 column (5 μm, 250 × 4.6 mm, ELITE) and a 210 nm ultraviolet (UV) detector. The column oven temperature was set at 30 °C. Linear gradient elution was used with water/0.05% trifluoroacetic acid (solvent A) and methanol/0.05% trifluoroacetic acid (solvent B) at 1 mL·min^−1^ and A/B ratios of 10:90, 100:0, 100:0, and 10:90 with run times of 0, 20, 23, and 25 min, respectively. For SAA preparation, the concentration of SAA was also determined by HPLC using an LC-2030 system equipped with a Hypersil ODS2 C18 column (5 μm, 250 × 4.6 mm, ELITE). The mobile phase was methanol:water:acetic acid (20:80:0.5, *v*/*v*/*v*), and the flow rate was set at 1.0 mL·min^−1^. The analyzing wavelength was 281 nm, and the column temperature was set at 30 °C [37].

## 4. Conclusions

Developing ecofriendly and high-efficiency methods for SAA production is an important challenge but is of great significance to best exploit the broad applications of SAA. In this study, we developed a novel, two-step biocatalytic reaction for the efficient synthesis of SAA from the inexpensive material of l-DOPA using whole-cell biocatalysts without the requirement of additional toxic reagents. Under the optimum reaction conditions, 48.3 mM of SAA could be prepared from 50 mM of l-DOPA, with a high yield of 96.6%. Based on these results, we conclude that the process developed herein is promising for the industrial production of SAA.

## Data Availability

The data presented in this study are available on request from the corresponding author.

## Supplementary Materials

The following supporting information can be downloaded at: https://www.mdpi.com/article/10.3390/molecules27186088/s1, The codon-optimized hydroxyphenylpyruvate reductase (HPPR) gene from *Coleus blumei*.

## Sample Availability

Samples of the compounds are not available from the authors.
