# Supplementary material for: Production of Salvianic Acid A from l-DOPA via Biocatalytic Cascade Reactions
Source: Molecules. 2022 Sep 18;27(18):6088. doi: 10.3390/molecules27186088 (PMC9501478; doi:10.3390/molecules27186088)
Supplement: Supplementary file 1 [file molecules-27-06088-s001.zip › molecules-1879174-supplementary.pdf]

Supplementary Materials

The codon-optimized hydroxyphenylpyruvate reductase (HPPR) gene from *Coleus blumei*:

GAAGCGATCGGCGTTCTGATGATGTGCCCGATGAGCACCTATCTGGAACAAGAACTGGATAAACG
TTTCAAACTGTTTCGTTATTGGACCCAGCCGGCACAGCGTGATTTCCTGGCGCTGCAGGCTGAAA
GCATCCGTGCGGTTGTTGGTAACTCTAACGCAGGTGCAGATGCGGAACTGATCGATGCTCTGCCG
AAACTGGAAATCGTTAGCTCTTTCTCCGTGGGCCTGGACAAAGTTGATCTGATTAAATGCGAAGA
AAAAGGCGTTCGCGTTACCAACACCCCAGATGTTCTGACCGATGATGTGGCGGATCTGGCAATCG
GCCTGATCCTGGCTGTTCTGCGCCGTATCTGTGAATGCGATAAATATGTGCGTCGTGGCGCGTGG
AAATTCGGCGATTTCAAACTGACCACCAAATTCTCTGGCAAACGCGTTGGTATTATCGGCCTGGG
CCGTATCGGTCTGGCAGTTGCGGAACGTGCGGAAGCGTTCGATTGCCCGATCAGCTACTTCAGCC
GTTCTAAAAAACCGAACACCAACTACACCTACTACGGCTCTGTTGTTGAACTGGCTTCTAATTCT
GATATCCTGGTTGTTGCTTGCCCGCTGACCCCGGAAACCACTCACATCATTAACCGTGAAGTTAT
CGATGCTCTGGGCCCGAAAGGCGTTCTGATCAACATCGGTCGTGGTCCGCACGTGGATGAACCGG
AACTGGTTTCTGCGCTGGTTGAAGGTCGTCTGGGTGGCGCTGGTCTGGATGTTTTCGAACGTGAA
CCGGAAGTTCCGGAAAAACTGTTCGGCCTGGAAAACGTTGTTCTGCTGCCGCACGTTGGCAGCGG
CACCGTTGAAACCCGTAAAGTTATGGCGGATCTGGTTGTTGGTAATCTGGAAGCGCATTTCAGCG
GTAAACCGTTGCTGACCCCGGTTGTTTAA
